# Mesenchymal stem cells in acute lung injury: are they ready for translational medicine?

**DOI:** 10.1111/jcmm.12063

**Published:** 2013-07-03

**Authors:** Feng Xu, Yue Hu, Jiebai Zhou, Xiangdong Wang

**Affiliations:** a Department of Respiratory and Critical Care Medicine Second Affiliated Hospital, Zhejiang University School of MedicineHangzhou, China; b Department of Respiratory Medicine Zhongshan Hospital, Fudan University Medical SchoolShanghai, China

**Keywords:** mesenchymal stem cells, acute lung injury, inflammation, infection, toxins

## Abstract

Acute lung injury (ALI) is a severe clinical condition responsible for high mortality and the development of multiple organ dysfunctions, because of the lack of specific and effective therapies for ALI. Increasing evidence from pre-clinical studies supports preventive and therapeutic effects of mesenchymal stem cells (MSCs, also called mesenchymal stromal cells) in ALI/ARDS (acute respiratory distress syndrome). Therapeutic effects of MSCs were noticed in various delivery approaches (systemic, local, or other locations), multiple origins (bone marrow or other tissues), or different schedules of administrations (before or after the challenges). MSCs could reduce the over-production of inflammatory mediators, leucocyte infiltration, tissue injury and pulmonary failure, and produce a number of benefit factors through interaction with other cells in the process of lung tissue repair. Thus, it is necessary to establish guidelines, standard operating procedures and evaluation criteria for translating MSC-based therapies into clinical application for patients with ALI.

- Introduction
- Complexity of ALI pathogenesis
- Therapeutic properties of MSCs in pre-clinical ALI models
- Potential Mechanisms of MSCs in ALI
  – Anti-inflammatory effect
  – Immunomodulatory effects
  – Reduction in pulmonary oedema
  – Clearance of bacteria
  – Lung repair
  – Microvesicles and mitochondria derived from MSCs
- Effects from MSCs or secondary reactions
- Considerations for translational medicine

## Introduction

Acute lung injury (ALI) as a severe pathological condition is characterized clinically by respiratory distress, refractory hypoxemia, and non-cardiogenic pulmonary oedema. A number of factors could initiate and cause the development of ALI, for example, sepsis, pneumonia, trauma, or aspiration of gastric contents [1], among which sepsis and pneumonia were accounted as the highest risk [2]. According to the American-Europe Consensus Criteria Joint Meeting of ALI diagnostic criteria, the annual incidence of ALI was about 78.9/100,000 in the United States in 2005 [3]. Patients with ALI/ARDS could not recover to normal predicted levels of physical function at 5 years and had greater 5-year costs for the incurrence of coexisting illnesses [4]. The mortality of severe sepsis with ALI caused by Gram negative or positive bacterial infection remains high [5, 6].

Efficient and specific therapies for ALI are still demanded because of the poor understanding of pathological mechanisms and limited effects of basic mechanical ventilation, fluid management, drug therapy to improve oxygenation, or other supportive methods [7]. Mesenchymal stem cells (MSCs, also called mesenchymal stromal cells) are currently proposed as a promising cell therapy for ALI, potentially attributed from a number of factors from MSCs *via* the paracrine and/or autocrine secretion [8–11]. This review aims to overview evidence of pre-clinical studies to support the application of MSCs for ALI, especially caused by bacterial infection or their toxins. We emphasized the importance of understanding the complexity of ALI from clinical aspects, molecular mechanisms by which MSCs exert their therapeutic effects and challenges to translate MSC-based therapies to clinical application.

## Complexity of ALI pathogenesis

**Fig. 1:**
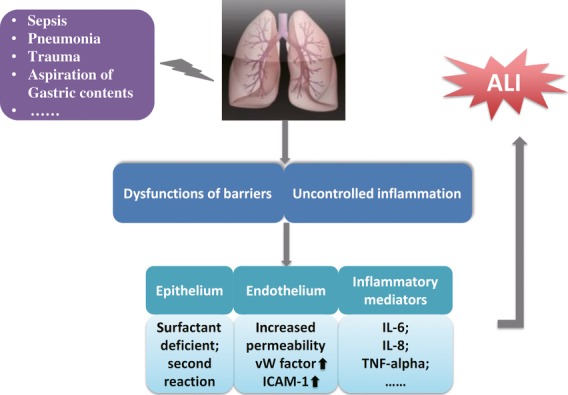
The pathogenesis of acute lung injury (ALI) is more complex than expected, which is characterized by uncontrolled inflammation and dysfunctions of endothelial and epithelial barriers of the lung.

Acute lung injury is characterized by uncontrolled inflammation and dysfunctions of endothelial and epithelial barriers of the lung, the loss of alveolar-capillary membrane integrity, excessive transepithelial leucocyte migration, and overproduction of pro-inflammatory mediators, as explained in Figure 1. The pathogenesis of ALI is more complex than expected. A number of biomarkers related with the lung epithelium and endothelium during the inflammatory and coagulation cascades were proposed to predict the morbidity and mortality of ALI [12]. The hyperproduction of inflammatory mediators, for example, interleukin (IL)-6, IL-8 and tumour necrosis factor (TNF)-α, has been considered as a direct response to injury. Protein C and plasminogen activator inhibitor-1 with alterations in coagulation and fibrinolysis are independent predictors of mortality [13]. Dysfunction of microvascular endothelial barriers results in the efflux of protein-rich fluid into interstitial tissue and distal airspaces of the lung, accompanied by increased release of von Willebrand factor and overexpression of intracellular adhesion molecule (ICAM)-1. In addition to the impairment pulmonary epithelial cells, for example, pneumocytes I and II, airway epithelial cells were also considered as the initiating and secondary reacting cells responsible for the development of acute lung inflammation and injury [14]. Recent studies also demonstrated that chemokines and their receptors play a dominant role in the initiation of leucocyte recruitment and lung inflammation [15]. Of multiple molecular mechanisms, the intracellular signal pathways like phosphoinositide 3-kinase (PI3K)-involved pathway have been suggested to control the signal transduction from the cellular membrane to the nuclei and regulate hyper-inflammatory responses of both leucocytes and epithelial cells [16].

Severe bacterial infection is recognized as the primary cause of ALI development, and the infected bacteria have been detected to release ciliary toxins, pneumolysin, endotoxin and IgA proteases, which compromise mucociliary clearance and activate dendritic cells, alveolar macrophages and epithelial cells through toll-like receptors (TLRs) to recognize pathogen-associated molecular patterns of the bacteria. Those activated cells could further produce a number of inflammatory mediators, for example, growth factors, chemokines, adhesion molecules and pro-inflammatory cytokines including IL-8 and TNF-α through nuclear factor (NF)-κB [17–19]. The bacteria-specific inflammation could simultaneously maintain host immune competence and facilitate repair.

## Therapeutic potentials of MSCs in ALI

**Fig. 2:**
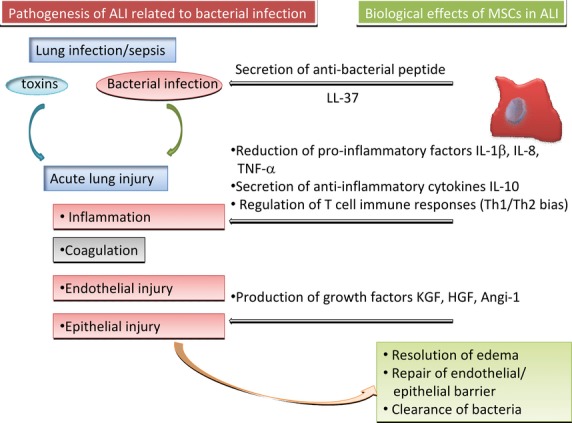
A promising way of MSCs-based cell therapy fits the therapeutic paradigm for acute lung injury (ALI)-biological effects and potential mechanisms with regard to the complicated pathogenesis of ALI.

Mesenchymal stem cells, discovered as adult multipotent cells in 1968 [20], are capable of self-renewing and differentiating into mesenchymal lineages, like chondrocytes, osteocytes or adipocytes [21], positive to CD105, CD90 and CD73, and negative to CD45, CD34, CD14 and CD11b [22]. Pre-clinical data showed therapeutic potentials of MSCs in ALI caused by endotoxin, pneumonia, or systemic sepsis, as summarized in Table 1 [11, 23–37]. The application of MSCs as a cell therapy for ALI was suggested to reduce lung inflammation, tissue oedema and injury, maintain the function of endothelial and epithelial barriers, accelerate the process of repair and to increase resistance to infection, as explained in Figure 2.

**Table 1:** Applying MSCs in pre-clinical ALI models caused by bacterial infection or toxins

ALI models	Resource and route of application of MSCs	Improved outcome	Ref.
IT LPS/rat	Intrapleural 1 × 10^6^ rat BM-MSCs with challenge	Lung injury, histology and inflammation	[11]
IT LPS/mice	IP 1 × 10^6^ human UC-MSCs 4 hr after challenge	Treg, survival time, body weight, histology and lung injury	[23]
OA LPS/mice	OA 2.5 × 10^5^ human BM-MSCs 4, 4.5 hr after challenge respectively	Inflammation and cytokines	[24]
CLP/mice	IV 2.5 × 10^5^ mouse BM-MSCs 6 hr after challenge	Mortality, injury, cytokine, and bacteria clearance	[25]
IT *E. coli*/mice	IT 1 × 10^6^ human BM-MSCs 4 hr after challenge	Bacterial clearance and inflammation	[26]
CLP/mice	IV 1 × 10^6^ mouse BM-MSCs 24 hrs before or 1 hr after challenge	Mortality and organ function	[27]
Endotoxin/*ex vivo* perfused human lung	Instil 5 × 10^6^ human MSCs 1 hr after challenge	Extravascular lung water, lung endothelial barrier permeability and alveolar fluid clearance	[28]
IP LPS/mice	IV 5 × 10^5^ mouse BM-MSCs 1 hr after challenge	Lung inflammation, injury and oedema	[29]
IT LPS/mice	IV 2.5 × 10^5^ mouse BM-MSCs with or without overexpressing angiopoietin 1 30 min. after challenge	Inflammation, cytokine and permeability	[30]
IP LPS/mice	IT 7.5 × 10^5^ mouse BM-MSC 4 hrs after challenge	Survival, and pulmonary oedema and permeability	[31]
IT endotoxin/mice	IV 2 × 10^7^ mouse bone marrow mononuclear cells 1 hr after challenge	Lung inflammation, alveolar collapse and interstitial oedema	[32]
IT *E. coli*/mice	IT 1 × 10^5^ human UC-MSCs 3 hr after challenge	Lung histology, inflammation and cytokine production	[33]
IP LPS/rat	IV 5 × 10^5^ human UC-MSCs 1 hr after challenge	Survival rate and inflammation	[34]
IT *E. coli*/mice	IT 7.5 × 10^5^ mouse BM-MSCs 4 hr after challenge	Survival and lung injury	[35]
IT LPS/mice	IV 3 × 10^5^ human orbital fat-derived MSCs 20 min. after challenge	Inflammation and permeability	[36]
IN LPS/mice	IT 2 × 10^5^mouse BM-MSCs 4 hr after challenge	Alveolar leucocytosis, protein leak, surfactant secretion and mortality	[37]

IV: intravenous; IP: intra-peritoneal; IT: intra-tracheal; CLP: caecal ligation and puncture; OA: oropharyngeal aspiration; IN: intranasal; LPS: lipopolysaccharide; MSCs: mesenchymal stem cells; UC-MSCs: umbilical cord MSCs; BM-MSCs: bone marrow derived-MSCs; ALI: acute lung injury.

There were 283 clinical trials registered for MSCs application (http://www.clinicaltrials.gov) and 45 studies for MSCs by December 2012, of which two were related to lung injury. One was in the phase II of clinical trial to test the safety and efficacy of human MSCs (PROCHYMAL™) for the treatment of moderate to severe chronic obstructive pulmonary disease (COPD), and another in the phase I to test the safety and feasibility of MSCs for treatment of emphysema with the current status of recruiting participants. However, there are no clinical trials registered for MSCs in ALI or ARDS, a serious form of ALI [38].

## Potential mechanisms of MSCs in ALI

### Homing and migration of MSCs

Mesenchymal stem cells could be migrated and homed with the involvement of chemoattractive cytokines, proteolytic enzyme, surface adhesion molecules and/or mesenchymal matrix metalloproteinases [32]. It was proposed that those signalling factors, including stromal cell-derived factor-1 (SDF-1), platelet-derived growth factor, hepatocyte growth factor (HGF), monocyte chemoattractant protein 1, basic fibroblast growth factor, or others [39], could be initially released by injured tissues and serve as messages for expansion of endogenous pools of MSCs or as local chemo-attractants for migration of implanted MSCs [40, 41]. On the other hand, MSCs could also express a number of chemokine receptors (CCR), for example, CCR1, CCR2, CCR4, CCR5, CCR7, CCR8, CCR9, CCR10, CXCR1, CXCR2, CXCR3, or CXCR5, in response to the chemokine-attractive gradients generated by the inflamed and/or injured site [39, 42, 43]. The communications between chemoattractive cytokines and their receptors enable MSCs to home diverse tissues, among which the SDF-1/CXCR4 axis was emphasized. SDF-1, a small chemoattractive cytokine designated as chemokine (C-X-C motif) ligand 12 (CXCL12), was up-regulated immediately after myocardial infarction and could sufficiently induce the migration of MSCs to injured sites [44]. SDF-1 could induce the expansion of MSCs through the rearrangement of actin cytoskeleton, polarization and phosphorylation of the focal adhesion adapter protein paxillin, and focal adhesion turn over and ultimately migration [41]. The functional expression of CXCR4 has been suggested to be required for the migration of MSCs to SDF-1 [45], and then the activation of the SDF-1/CXCR4 axis could enhance the migration of MSCs following bleomycin-induced lung injury [46].

It is possible that the communication and interaction between adhesion molecules on MSCs and endothelial cells in compromised tissues may orient MSCs to the targeted tissue. Adhesion molecules have been suggested to play directional role in MSC homing. For example, MSCs could interact with pulmonary endothelial cells directly and maintain the integrity of endothelial barrier by preserving adherens junctions (VE-cadherin and β-catenin) and inhibiting leucocyte adhesion and adhesion molecule expression (VCAM-1 and ICAM-1), demonstrated by both *in vitro* and *in vivo* studies [38]. Intravenously administered MSCs were found to have more affinity to the lung and liver in endotoxemia induced by LPS [47], and more MSCs appeared in the lung, kidney, and spleen in response to polymicrobial sepsis [27]. These findings indicate that the lung is one of the most predominant organs/tissues for MSC homing, even though they could also home to and appeared at diverse sites of injury during the development of the multi-organ injury and dysfunction accompanied by the existence of ALI.

### Anti-inflammatory effects

Acute inflammation is one of important defences against infections and foreign toxins, whereas uncontrollable inflammation caused by sepsis can compromise the lung and other organs [48]. MSCs were found to protect the host from extraordinary inflammatory damage by enhancing host resistance to sepsis, and down-regulate expressions of pro-inflammatory factors, for example, IL-1β, IL-8, interferon (INF)-γ, TNF-α or others [49]. MSCs could also regulate the development of lung inflammation through the production of anti-inflammatory agents such as IL-4 and IL-10 [50], the reduction in neutrophil infiltration into the lung and attenuation of the pro-inflammatory cytokines in circulation, to maintain the balance of inflammatory and anti-inflammatory responses [51]. The therapeutic effects of MSCs on *E. coli*-induced lung inflammation were demonstrated by the intratracheal transplantation of MSCs 3 hrs after the instillation of bacteria. Such ‘treatment’ could increase survival, attenuate lung injuries and reduce leucocyte infiltration and production of IL-1α, IL-1β, IL-6, TNF-α and macrophage inflammatory protein (MIP)-2, 3 days after the induction of ALI in mice [33]. A recent study demonstrated that human umbilical cord-derived MSCs could ameliorate LPS-induced ALI in a rat model, and found that those MSCs increased survival rate and reduced circulating levels of TNF-α, IL-1β and IL-6, but not IL-10 [34]. MSCs could ameliorate the severity of lung inflammation, oedema, or injury. It may be a smart strategy to use MSCs as a temporary solution to down-regulate inflammatory responses and tissue injury in such acute phase.

### Immunomodulatory effects

Mesenchymal stem cells could suppress or modulate innate and adaptive immune responses [52], probably through inhibiting the activation and proliferation of T lymphocytes and affecting functions of a broad range of immune cells, including nature killer (NK) cells, B cells and dendritic cells [53]. MSCs could switch Th1 cell-based inflammatory response to Th2 cell-based anti-inflammatory response during the inflammation, probably through the dependent and/or independent interactions between inflammatory cells. Such interaction could lead to the release of soluble factors, including transforming growth factor (TGF)-β, prostaglandin (PGE) 2, IL-10 and IL-1RA. It was suggested that MSCs could be involved in the initiation of anti-inflammatory effects and immunosuppression through the expression and regulation of TLRs [54]. The immunosuppressive phenotype of human bone marrow-derived-MSC was enhanced by activation of TLR3 and TLR4, dependently on the production of immunosuppressive kynurenines by the tryptophan-degrading enzyme indoleamine-2,3-dioxygenase-1 [55]. The ligation of TLR3 and TLR4 could inhibit the T cell modulatory ability of MSC by impairing Notch signalling, without influencing the differentiation potential [56]. In addition, the ‘danger’ signals could be recognized through TLRs, which are activated and triggered to mobilize innate and adaptive host immune cells through the recruitment of MSCs [57]. MSCs have more complicated effects on the immune system than the classical role as immune suppressor cells. It will be important to understand the mechanisms by which MSCs play apparently paradoxical roles in the immune response for developing MSC-based therapy in clinical application.

### Attenuation of pulmonary oedema

The disruption of the lung microvascular endothelial cell barrier results in the increased lung vascular permeability and plasma extravasation, inflammatory cell infiltration, tissue oedema and injury, as well as impairment of gas-blood exchange [31], whereas therapy of MSCs could markedly attenuate pulmonary oedema in ALI [58, 59]. One of the potential mechanisms by which MSCs could prevent or treat lung oedema in ALI is that a number of growth factors from MSCs may maintain or cure the endothelial cell barrier through the initiation of the healing and repair process through the binding to correspondent receptors on local cells. For example, MSCs can produce several growth factors, including keratinocyte growth factor (KGF), HGF, angiopoietin-1 and fibroblast growth factors. Of them, KGF could reduce pulmonary oedema in animals with ALI partly through increasing activities of epithelial sodium channels and Na-K ATPase and capacities for alveolar fluid transport [60–62]. KGF induced alveolar type II cell proliferation through PI3K and p42/44 MAP kinase pathway [63]. HGF could reduce pulmonary oedema through T-iam1-mediated Rac and Rho GTP binding proteins pathways [64, 65], whereas angiopoietin-1 might prevent the dysfunction of the endothelial barrier and inhibit the interaction between leucocytes and endothelial cells by modifying endothelial cell adhesion molecules and cell–cell junctions [66]. In addition, implanted MSCs were proposed to directly integrate with damaged lung tissue, repair alveolar-capillary membrane, reduce alveolar-capillary membrane permeability, and improve diffuse pulmonary oedema [30, 32, 59].

### Clearance of bacteria

Mesenchymal stem cells were found to improve the phagocytosis of resident immune cells to reduce bacterial burden and to increase survival of macrophages by up-regulating associated genes [54, 67]. Peptides produced from MSCs could directly have the anti-bacterial activity, demonstrated by the finding that cathelicidin LL-37 produced by MSCs increased in experimental ALI caused by bacterial challenge and had antimicrobial effects by disrupting bacterial membranes [26, 68]. Intratracheal treatment with wild-type MSCs improved the lung injury and survival in animals with bacterial pneumonia, and enhanced bacterial clearance from the alveolar space 4 hrs after administration, partly because of the upregulation of the antibacterial protein lipocalin 2 [35]. Intratracheal transplantation of umbilical cord blood-derived MSCs was found to attenuate *E. coli*-induced ALI primarily by down-modulating the inflammatory process and enhancing bacterial clearance [33]. It implies that the enhanced clearance of bacteria by MSCs may contribute to the improvement of ALI induced by Gram-negative bacterial infection, or MSCs had directly inhibitory effects on the growth of Gram-positive bacteria [26].

### Lung repair

Mesenchymal stem cells play an important role in the repair process, which may be initiated simultaneously with the development of ALI and may differentiate into lung epithelial cells and directly replace damaged cells in alveoli during the course of ALI. A single bone marrow-derived MSC could differentiate into cells of multiple different organs/tissues, of which 20% grew in the lung and even differentiated into alveolar epithelial cells [69]. Bone marrow-derived stem cells engrafted into pulmonary epithelial layer and exhibited specific characteristics of lung epithelial cells [70]. Pulmonary epithelial cells could be originated from circulating progenitors of both bone marrow and circulation identified by CK5^+^CXCR4^+^ markers [71]. CXCR4/CXCL12-mediated recruitment of circulating progenitor epithelial cells was believed to be necessary for the reestablishment of a normal pseudostratified epithelium after airway injury. However, some studies on experimental lung injury demonstrated the engraftment rate of MSCs was less than 1–5% [31, 52, 69]. MSCs enhanced restoration of systemic oxygenation and lung compliance, reduced total lung water, lung inflammation and histological lung injury, and restored lung structure in ventilation-induced ALI, probably through MSC-secreted products rather than MSCs *per se*. Such therapeutic effects on lung repair were suggested to be a KGF-dependent paracrine mechanism [72].

## Microvesicles and mitochondria derived from MSCs

Increasing evidence has suggested a critical role of microvesicles in cell-based therapies in ALI and other disease models [37, 73–75]. Studies from Bruno *et al*. demonstrated the microvesicles isolated from human adult MSCs could effectively protect against acute tubular injury [74] or ischaemia–reperfusion-induced acute and chronic kidney injury [75]. Microvesicles conferred the resistance of tubular epithelial cells to apoptosis and stimulated proliferation, and the effect of microvesicles on the recovery of acute kidney injury was similar to the effect of human MSCs [74, 75].

Microvesicles, as small circular membrane fragments, are shed from the cell surface or released from the endosomal cell membrane compartment and play an important and underappreciated role in cell-cell communication [73]. The intriguing microvesicle-mediated cell–cell communication system emerged very early during evolution and served as a template for the further development of intercellular interaction mechanisms involving soluble bioactive mediators and fine-tuned ligand-receptor interactions. The microvesicles may transfer specific genes or small organelles from MSCs to the injured targets to exert the protective effects. Most recently, these microvesicles have been shown to mediate the transfer of mitochondria, derived from bone marrow-derived stromal cells, into the alveolar epithelium in a LPS-induced ALI model [37]. The stromal cells formed connexin 43-containing gap junctional channels with the alveolar epithelia, releasing mitochondria-containing microvesicles that the epithelia engulfed. These gap junctional channels and transferred mitochondria appeared to play a pivotal role in mediating the therapeutic effects of the stromal cells, as LPS-induced ALI was markedly abrogated by the instillation of wild-type stromal cells, but not of cells with gap junctional channel-mutant or with dysfunctional mitochondria [37]. Microvesicles act as important mediators of intercellular communication and exert important biological effects on target cells, rather than cell debris or biologically irrelevant cell dust. Application of MSC-derived microvesicles to ALI appears to be a potential approach.

## Effects from MSCs or secondary reactions

Mesenchymal stem cells *per se* were proposed to have therapeutic effects on ALI by the proliferation and differentiation in the lung, although the production of mediators from MSCs was considered to be even more dominant in the protective mechanisms. It was found that MSCs could down-regulate pro-inflammatory responses to endotoxin and up-regulate the production of anti-inflammatory cytokines in the lung after an intrapleural administration of MSCs [11]. The injected MSCs were traced and found to be located in the intrapleural site rather than in lung tissue, indicating that the therapeutic effects of MSCs may result from the production of factors from MSCs *via* a paracrine/endocrine mechanism. Intravenous injection of orbital fat-derived stem/stromal cells could reduce LPS-induced pulmonary inflammation, endothelial and alveolar epithelial permeability, infiltration of neutrophil and macrophage, systemic proinflammatory chemokine levels such as macrophage inflammatory protein-1-γ, B-lymphocyte chemoattractant, IL-12 and subsequent circulation helper T cell numbers, whereas few human orbital fat-derived stem/stromal cells were detected in the recipient lung after acute inflammation subsided [36].

## Considerations for translational medicine

Mesenchymal stem cells-based cell therapy is proposed as one of promising therapies for ALI. Abundant pre-clinical studies helped us to understand the pathogenesis of ALI and the mechanisms of MSCs for the therapeutic potential [11, 23–37]. However, a number of issues should be considered and explored before MSCs become a viable therapy for ALI, including therapeutic efficacy, dosage, starting time, duration, frequency, standard, and monitoring.

A broad spectrum of tissues, including bone marrow, adipose tissue, muscle, dermis, placenta, or lung, have been identified as resources for MSCs, of which the lung stem and progenitor cells as an endogenous resource were found to be similar to MSCs in the bone marrow [40, 41]. Among them, the bone marrow is commonly considered as the major origin of MSCs for experimental ALI, however, adipose-derived stem cells (ADSCs) should not be neglected as one sort of the most popular adult stem cells which have been used to treat various diseases in both pre-clinical research and clinical trials [76]. The potential effects of ADSCs on ALI will attract more attention and should be further evaluated in future studies. The roles of lung endothelial progenitor cells or vascular endothelial progenitor cells as resources of MSCs need to be further explored too. The predominant migration of MSCs to injured tissue can be used to design therapeutic strategies, for example, to apply MSCs as vectors for delivering pharmacological vehicles into the injured lung or to improve cell-based therapy *via* modulating MSCs migration.

The therapeutic effects of MSCs on ALI are still questioned [77], even though a number of studies on pre-treatment with MSCs have been performed in mice, rats, or rabbits. The reason for the existence of such questions and concerns is that there is still lack of evaluation criteria for the success of MSCs treatment, standardized manipulation of MSCs preparations, studies designed with a large scale of animals or performed in even large animals like pigs, or clinical phenotypes and outcome measured. It is also questionable how far MSCs can be applied to treat patients with ALI/ARDS, what critical steps are to be used to translate pre-clinical trials into clinical therapies, and whether MSCs are the hope or hype for clinical application [49]. It is necessary to clarify whether MSCs *per se* or MSCs-associated factors play the most important role in the prevention and therapy for ALI/ARDS, which leads to different translational strategies of cell-based therapy to clinical application. Logical strategies and designs of pre-clinical studies to tackle those questions should be well considered and carried out. For example, MSCs-derived materials such as cell culture supernatants or microvesicles may be used to mimic the effects of MSCs in various disease models [26, 37, 73–75]. Furthermore, defined target molecules from MSCs with obvious therapeutic efforts should be screened and selected through targeted proteomics and systems biology.

In conclusion, there is increasing evidence from pre-clinical studies to support preventive and therapeutic effects of MSCs in ALI/ARDS. Therapeutic effects of MSCs were noticed in various deliveries of cells (systemic, local, or other locations), multiple origins (bone marrow or other tissues), or different schedules of administrations (before or after the challenges). MSCs not only reduced the over-production of inflammatory mediators, leucocyte infiltration, tissue injury and pulmonary failure but also produced a number of beneficial factors and, through interaction with other cells, participated in the process of lung tissue repair. There are urgent needs of strategies, guidelines, standard operating procedures to MSC handling and evaluation criteria of patient recruitment and therapeutic efficacy to translate MSCs-based therapies into clinical application, a possible new and effective approach against ALI. Thus, we have to seriously consider if it is the time that MSCs are ready for clinical trials and applications or we are too optimistic to translate MSC therapy into clinical applications.
